# Energy-Efficient IoT Service Brokering with Quality of Service Support †

**DOI:** 10.3390/s19030693

**Published:** 2019-02-08

**Authors:** Giacomo Tanganelli, Enzo Mingozzi

**Affiliations:** Department of Information Engineering, University of Pisa, L.go Lazzarino 1, I-56122 Pisa, Italy; enzo.mingozzi@unipi.it

**Keywords:** Internet of Things (IoT), quality of service, resource management, energy-aware allocation, real-time systems

## Abstract

The Internet of Things (IoT) is becoming real, and recent studies highlight that the number of IoT devices will significantly grow in the next decade. Such massive IoT deployments are typically made available to applications as a service by means of IoT platforms, which are aware of the characteristics of the connected IoT devices–usually constrained in terms of computation, storage and energy capabilities–and dispatch application’s service requests to appropriate devices based on their capabilities. In this work, we develop an energy-aware allocation policy that aims at maximizing the lifetime of all the connected IoT devices, whilst guaranteeing that applications’ Quality of Service (QoS) requirements are met. To this aim, we formally define an IoT service allocation problem as a non-linear Generalized Assignment Problem (GAP). We then develop a time-efficient heuristic algorithm to solve the problem, which is shown to find near-optimal solutions by exploiting the availability of equivalent IoT services provided by multiple IoT devices, as expected especially in the case of massive IoT deployments.

## 1. Introduction

The Internet of Things (IoT) has rapidly evolved from a cutting-edge research topic to a real ecosystem affecting people’s everyday life. As a matter of facts, smart cities, smart factories and smart homes are concepts already familiar even to non-technicians. Nevertheless, we are still at the beginning of the IoT era. According to a new analysis from IHS Markit [1], the number of connected IoT devices will grow from nearly 27 billion in 2017 to 125 billion in 2030. Such massive increase will require robust and complex management systems, which are still in their infancy. Smart cities, for example, are nowadays characterized by many different isolated IoT systems, e.g., *smart parking systems, smart transportation, etc.*, where each system requires its separated management infrastructure. With the growing of the IoT domain, the development of a comprehensive IoT architecture, which will be able to integrate heterogeneous IoT systems, is of paramount importance. 

Recently, some efforts have been carried out in this direction, and different international organizations ad industries are developing standard architectures for IoT. We can envision two different approaches. The first one extends cloud-based commercial platforms in order to integrate IoT systems, with many available solutions such as Amazon AWS IoT (https://aws.amazon.com/iot-core/), Google IoT (https://cloud.google.com/iot-core/) and Microsoft Azure IoT (https://azure.microsoft.com/en-us/services/iot-hub/) platforms. The main idea of these IoT-Cloud architectures is to integrate IoT devices directly into the cloud infrastructure, which is exploited to interconnect different IoT systems in order to provide a unified interface to end users on one side, and to exploit the capabilities of the cloud, in terms of storage and computation, on the other side. Such architectures are the solid ground for the realization of service-brokering solutions by providing a centralized point of control between smart things and applications. The second approach, instead, is based on the definition of novel architectures that can exploit the unique characteristics of IoT systems by design. Among the others, the oneM2M (http://www.onem2m.org/) and the FIWARE (http://www.fiware.org) architectures are getting momentum. The former was originally designed by the European Telecommunications Standards Institute (ETSI), and it is specifically tailored to machine-to-machine (M2M) communications. FIWARE, instead, is a generic framework where different components, called Generic Enablers (GEs), can be assembled together in order to create an integrated service platform. By means of a specific GE, called IDAS (https://catalogue-server.fiware.org/enablers/backend-device-management-idas), the FIWARE architecture can also be used as an IoT platform. The current trend is therefore the integration of smart things into a common IoT platform that, in turn, exposes *thing* capabilities as separate services. Applications interact with things by invoking IoT services exposed by the platform. 

Especially in massive IoT deployments, the huge number of connected *things* allows applications to benefit from high IoT service availability: different *things* may provide *equivalent* services but with different QoS and cost (e.g., different smart cameras may provide, if appropriately steered, the same view of a given area from different directions). To handle this, many different IoT-Cloud solutions, such as [2,3,4], expose to users virtual sensors which are resolved to physical sensors at runtime, achieving the so-called sensing-as-a-service solution. However, *things* are constrained devices in terms of computation, storage and energy. Things are also more volatile and dynamic: a continuously changing context and intermittent availability are the two main factors that differentiate IoT services supported by smart things from traditional services running on fixed powerful servers. Therefore, novel service brokering approaches and service allocation algorithms are needed, which are capable to address the unique characteristics of smart things while guaranteeing to meet the applications’ QoS requirements.

In this work, we focus on the definition of a thing allocation policy that aims at maximizing the lifetime of all the connected *things*. Our contribution is twofold: (i) an energy aware service selection problem is formally defined as a non-linear generalized assignment problem (GAP), and (ii) a time-efficient heuristic algorithm, which finds a solution in a time suitable for implementation in real systems, is developed and evaluated. The proposed solution is, therefore, agnostic to the underlying communication layer and can be easily deployed in almost any available IoT platform, e.g., FIWARE. The rest of the work is organized as follows. In Section 2 we report the works relevant to our problem. A detailed presentation of the considered scenario is reported in Section 3, whereas the derived assignment problem is formally presented in Section 4. The heuristic algorithm is presented in Section 5 and evaluated in Section 6, respectively. Finally, Section 7 draws the conclusions. 

## 2. Related Work

The QoS-aware allocation problem has been investigated in different fields, each one characterized by different requirements and guarantees. In Service Oriented Architecture (SOA), the main challenge is to consider the requirements of both service providers and clients in order to select the correct service among a known set [5]. In [6] authors propose a QoS broker that exploits a utility function to combine services in order to maximize the requirements of the users. However, the proposed solution does not consider provider constraints. In [7], instead, the authors try to put in the loop also the provider constraints. The solution is based on a dynamic load balancer strategy that, however, allows to express requirements only in terms of fixed priorities. A different approach is presented in [8], where the authors propose a heuristic algorithm to find the optimal allocation minimizing the overall average response time. The problem is that, in the IoT domain, probabilistic guarantees are not always a feasible choice. Moreover, the authors do not consider energy requirements, which are of paramount importance when we deal with large IoT systems. Multiple QoS parameters are addressed instead in [9]. The work focuses on the selection of services among a huge set of candidate services, each one characterized by different QoS parameters. The selection is performed combining probabilistic QoS estimations to reduce the possible candidate services, with integer programming, to find the ‘optimal’ solution within the reduced set. This solution, however, does not consider specific IoT requirements, such as the computational time needed to execute a service, and it is more tailored to service composition rather than IoT service selection.

From the previous analysis we can conclude that QoS-aware solutions designed for Web Services cannot be directly mapped to the IoT domain due to different constraints. More specifically, in this context services are provided by IoT devices that are constrained in terms of computation and communication, the availability of a service is affected by the energy of the IoT device, and the context information are derived from the physical environment. Constrained scenarios and energy concerns are however analyzed by solutions designed for Wireless Sensor Networks (WSNs), in which task assignment has been a heavily studied problem. In [10] the authors present two algorithms, one centralized and one distributed, to distribute tasks among sensor nodes in order to maximize the network lifetime. The solution, however, is tailored to WSNs characterized by a sink, which collects data from nodes, and cannot be directly mapped to a generic IoT scenario. The same problem is also addressed in [11], where the authors develop a greedy algorithm to minimize the energy consumption in networks composed by heterogeneous sensor nodes. As in our solution, the authors exploit the heterogeneity of energy costs to assign tasks. However, the algorithm relies on a deep knowledge of the underlying communication infrastructure, i.e. direct connections between sensor nodes, which is not always feasible in IoT systems that are usually agnostic to the network layer. 

QoS-aware task allocation has been studied also in the field of task scheduling on multiprocessors. Specifically, the problem can be seen as a problem of multi-processor partitioning in which all service requests are independent tasks. In [12] the authors developed a polynomial time algorithm to partition a set of sporadic tasks among multiple processors. However, the proposed solution assumes that all processors have the same capabilities. Heterogeneity of processors is addressed in [13], however only the CPU utilization is considered, without evaluating the energy consumption of each assignment. Finally, in [14] the authors proposed a solution to solve the partitioning problem, taking into account also energy constraints, in scenarios characterized by heterogeneous processors. The work, however, leverages voltage-varying processors, that have not a reasonable correspondence in the IoT domain. 

In [15] the authors propose a QoS-aware scheduling algorithm designed for service-oriented IoT platforms. A multi-layered scheduling model is proposed to evaluate the optimal allocation that meets the QoS requirements of applications. Different solutions are deployed at different layers to manage different system resources, such as network resources and IoT services. However, the proposed approach cannot be used for online IoT-service selection, since it is mainly suitable for offline provisioning and planning. The authors of [16] addressed the service selection problem in the IoT environment by developing an energy-centered QoS-aware service selection algorithm, called EQSA. As stated by the authors, the basic idea was to preserve energy by slightly reducing the QoS level without affecting the user’s satisfaction. To this aim, a multi-objective optimization problem is solved by means of lexicographic optimization strategy, which takes into account multiple QoS parameters ranked by order of importance. As in our proposed solution, the selection algorithm exploits *equivalence* among services by means of a differentiation between abstract and concrete services. However, the work in [16] focuses more on service composition and each abstract service is finally mapped to one and only one concrete service. 

The service selection problem has been considered also within IoT-Cloud architectures, in order to reduce the energy consumed by connected physical sensors. In [2,3], users’ requests are aggregated based on the resolved physical sensors in order to save energy. Moreover, in [2] the typical periodic sensing model is replaced in favor of an on-demand interactive pattern, driven by location information, which reduces the energy consumed by each physical sensor. Indeed, sensors are clustered by means of regions of interest, and each request is resolved by the sensor within the cluster having the highest residual energy, whereas the other nodes of the cluster are left in sleep mode. Similarly, in [3] the IoT-Cloud paradigm is exploited to provide latency guarantees to applications interacting with the same physical sensors. A dedicated QoS controller is deployed within the cloud infrastructure to aggregate requests for virtual sensors and to set the periodic sampling rate, on physical sensors, accordingly to the most demanding application. In this way, the overall workload is minimized and the duty-cycle of connected sensors can be dynamically changed in order to save energy, whilst the latency requirements are guaranteed. Differently from our solution, however, all the previous work focused mainly on WSNs characterized by a tree topology rooted at a sink, where the cost associated to duty-cycles are of paramount importance. In addition, the computational cost on each thing is addressed only marginally.

A further step has been recently achieved by means of the integration of the fog layer between the cloud and the smart devices. As a matter of fact, in [17] the authors propose a sensing-as-a-service architecture that exploits geographically distributed “cloud agents” to interconnect cloud users to remote IoT devices by means of virtual sensors. Each user issues sensing tasks for specific virtual sensors enriched also by means of some QoS parameters that are enforced by the cloud agents. The fog layer is also exploited in [18] to partition resources among interconnected fog nodes in order to reduce services’ latency. Services are partitioned among fog nodes based on popularity metrics. However, the work does not consider costs on the connected smart devices. Similarly, in [19] the authors presented an exhaustive survey on the state of the art techniques for service placement between the fog layer and the cloud, highlighting pros and cons of each presented solution. Also in this case, the focus is primarily on the placement of computational resources between the cloud and the fog, whereas smart devices, with their unique characteristics, are not directly considered. Finally, in [20] the fog layer is exploited to provide additional delivery mechanisms to users. Indeed, the authors presented a multi-method data delivery system that exploits the most convenient delivery mechanism based on the actual location of the user. Specifically, WSNs are connected to a cloud environment, which is then exploited by users and fog servers. A user may retrieve the same information from four different data sources: Cloud, WSN, other users, or fog servers. The selection is based on an objective function that minimizes the cost or the delivery time. 

It is worth to note that, even in IoT-Cloud solutions, the mapping between virtual and physical sensors is always one-to-one. The first solution that allows to map a virtual sensor to multiple physical sensor has been presented in [21]. Specifically, a Sensor-Cloud infrastructure is presented that allows mapping virtual sensors to physical sensors following one-to-many, many-to-one and many-to-many patterns. Moreover, the cloud is exploited to allow data aggregation and to set the sampling rate according to the most demanding application. Energy consumption on sensor nodes is however addressed only marginally: QoS parameters, expressed by the users, focus on data accuracy and reliability. The mapping of requests to different services is also addressed in [22], where the authors focus on the allocation of resources on IoT devices characterized by multiple network interfaces. The requests are assumed to be flexible in the sense that they can be realized by splitting their demands on multiple interfaces, thus achieving a one-to-many mapping. The authors propose a MILP formulation, which also considers different costs per interface and two algorithms to approximate the optimal solution. The problem considered is however tailored to the specific use case characterized by few interfaces (less than 10), and the split of requests is performed only when not enough resources are available on a single interface. 

Finally, in our previous work [23], we addressed the scheduling of real-time requests in the IoT domain by developing a greedy polynomial-time algorithm that can be used to allocate requests to things taking into account real-time requirements of applications with strict deadlines. Our proposed approach, however, was tailored to periodic requests with implicit deadlines and was designed to assign a request to a single thing, i.e., we did not allow the execution of the same request among different things on each period. In this work, we go a step further by allowing requests with a deadline larger than the period, and by fully exploiting service equivalence in order to further distribute the energy cost more uniformly among all the connected things.

## 3. Reference Architecture

**Figure 1:**
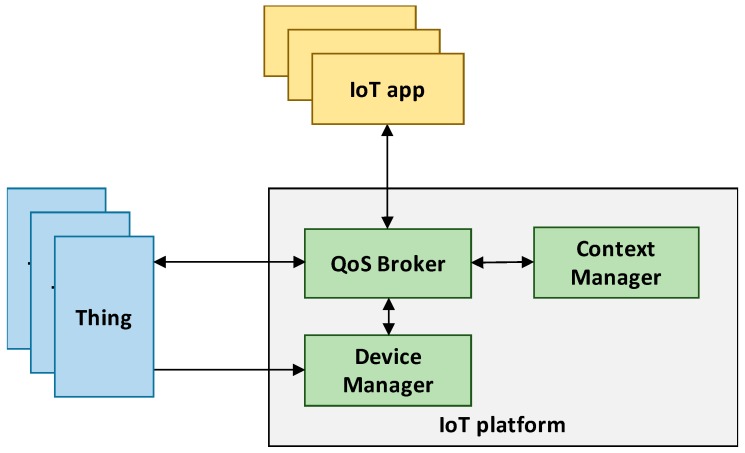
Reference architecture.

In Figure 1 we report the reference architecture considered in this work. We focus on a massive deployment of constrained IoT devices managed by an IoT service platform. Each IoT device has sensor and/or actuator capabilities that can be accessed as a service (e.g., through a RESTful interface). We generically refer to such IoT devices as smart *things*. Moreover, we assume that IoT services provided by things are context-aware, i.e., they are further characterized by context information. Context, defined as “any information that characterize the situation of an entity” [24], enhances the description of a service, e.g., measure the temperature, with any additional relevant information related to it, like, e.g., its location or its freshness. 

Given the very large number of connected things, it is expected that multiple things may possibly provide the same or equivalent services, and the platform is able to exploit such redundancy. However, though two things expose the same service, context information characterizing the two service instances might differ. For example, if an application looks for the temperature in a specific geographic area, only *things* equipped with a temperature sensor and deployed in the interested area (as resulting from the attached context information) may be consistently selected to reply. In fact, an application interacts with the platform by requesting a specific IoT service matching a given context information (e.g., only if provided by things located in a specific area) and meeting certain QoS requirements. The IoT platform must then map each application request to specific services exposed by connected things. In order to develop a generic framework that can be exploited by generic IoT platforms regardless of the communication network employed by the connected things, we assume that the IoT platform implements three main functional components: (i) a *Device Manager*, (ii) a *Context Manager*, and (iii) a *QoS Broker*. The Device Manager handles information about the status of all the connected things, e.g., computational, communications, storage capabilities, as well as the amount of residual energy if battery-powered. The Context Manager, instead, handles all context information related to services provided by things, and provides a context-based service look-up functionality. Specifically, the Context Manager processes each application request and, based on the context information associated to each thing service, it produces a list of *equivalent services* that meet the context requirements specified by the request. Please note however, that algorithms for context management and analysis are outside the scope of this work.

Finally, the QoS Broker is the component in charge of allocating requests to things. Based on the status information provided by the Device Manager and the list of *equivalent services* provided by the Context Manager, the QoS Broker verifies if all requests can be satisfied. It then allocates each request to at least one thing according to a dedicated *resource allocation policy*. It is worth to note that the considered broker-based architecture is agnostic to the underlying things’ communication network and can be easily mapped to multiple real IoT platforms available today. As an example, the core of the FIWARE platform is the Orion Context-Broker, which manages all context information and, among the other offered services, it allows applications to perform context queries to interact with available services. 

**Figure 2:**
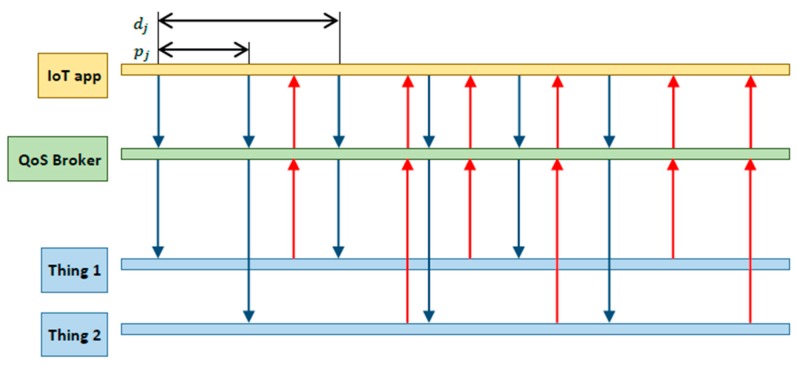
Periodic service invocations.

We focus, in this work, on the definition of a resource allocation policy for the QoS broker. The aim of the policy is to maximize the lifetime of the IoT platform while meeting the QoS requirements specified by applications. To achieve that, the proposed policy is allowed to exploit the availability of multiple *equivalent* services by possibly mapping (i.e., splitting) an application request to multiple things. As for the QoS requirements, we assume that an application request is conveyed as a sequence of real-time periodic service invocations with time-bounded requirements – see Figure 2. Therefore, a request is characterized by a period and a relative deadline. We assume that each deadline can be equal or larger than the corresponding request period. A period smaller than the relative deadline means that the application is willing to issue service invocations at a rate larger than the expected one at which a single thing is able to issue a response, thus implicitly assuming that its request needs to be split to multiple things in parallel. To better clarify this, let us consider the following example. Assume that a request has an associated period of 5 s and a relative deadline of 10 s. At time t = 0, the QoS broker will map the first service invocation to a thing able to provide the requested service in 7 s. At time t = 5, the second service invocation is issued, but the QoS broker has to select a different thing to serve it, since the previous one is still in the processing phase. Therefore, two service instances will be running in parallel on different things to serve the same application request. The application will receive two responses, each one before its corresponding deadline. 

Finally, as mentioned, the aim of the proposed policy is to maximize the lifetime of the platform. In fact, the execution of each service on a thing requires a certain amount of energy. If the thing is battery powered, its capability to provide an IoT service has a maximum lifetime that depends on the number of periodic service invocations. The lifetime of the overall IoT platform is therefore determined by the thing with the shortest lifetime. The proposed policy aims at maximizing the latter by carefully mapping application requests to things, eventually splitting one request to multiple things so as to evenly balance the energy cost of execution among all things.

## 4. Problem formulation

We now formally define the allocation problem addressed in this work. We assume the IoT deployment managed by the platform comprises a set N of n things, where each thing i∈N is characterized by its available energy $b_{i}$, (possibly equal to +∞, if not energy-constrained).

Based on the capabilities of the managed things, the platform provides a set of IoT services requested by client applications. We assume that there is a set K of k application requests that must be accommodated by the platform. Specifically, each request j∈K is characterized by a period $p_{j}$ and a deadline $d_{j}$, with $d_{j}\geq p_{j}$.

Not all things can serve the same request, but the same request can be served by multiple things. We denote things’ service capabilities by means of a *context matrix* M where each element $m_{ij}\in M$ can be zero or one depending if the thing i can serve the request j or not:

(1) $$m_{ij}=\{\begin{array}{c} 1\;\mathrm{if}\;\mathrm{request}\;j\;\mathrm{can}\;\mathrm{be}\;\mathrm{served}\;\mathrm{by}\;\mathrm{thing}\;i\\ 0\;\mathrm{otherwise} \end{array}$$

Without loss of generality, we assume that each service request can be executed by at least one thing, i.e., for any j, there is at least one i such that $m_{ij}=1$.

We model the costs of execution of a request j on a thing i by means of two parameters: the *execution time* $t_{ij}$ and the *energy cost* $c_{ij}$. The execution time $t_{ij}$ denotes the amount of time needed to execute one service invocation of request j on thing i, including both communication and computation times, whereas $c_{ij}$ represents the amount of energy needed to accomplish the execution of one service invocation of the request j on thing i.

We further define the *utilization matrix* (U) and the *energy consumption rate matrix* (F), respectively. Specifically, the utilization is defined as $u_{ij}\in U$:

(2) $$u_{ij}≜\{\begin{array}{c} \frac{t_{ij}}{p_{j}}\;\;\;if\;m_{ij}=1\\ +\infty \;\;\;\mathrm{otherwise} \end{array}$$

where we set an infinite utilization in case $m_{ij}=0$, in order to filter out things that cannot provide the requested service.

Moreover, in order to make a fair comparison among things, we normalize the energy cost of execution $c_{ij}$ with respect to the available energy $b_{i}$, and we then define the *relative energy consumption rate* $f_{ij}\in F$ of executing the request j on thing i as:

(3) $$f_{ij}≜\frac{1}{p_{j}}\frac{c_{ij}}{b_{i}}$$

As already pointed out, we assume the platform is able to exploit the *equivalence* among services in order to extend the lifetime of the connected things. Specifically, we allow service invocations of a request to be mapped (i.e., split) to multiple things across consecutive periods. As a consequence, each thing selected to serve a certain request j will serve a service invocation with a period $s_{j}$ times larger than the request’s period $p_{j}$, thus the energy and utilization costs to execute the request per thing is reduced. We call the number of things involved in the execution of a certain request j the *split factor* $s_{j}$ of request j. The *split factor* $s_{j}$ is enforced to be upper-bounded by $s_{j}^{max}$ defined as:

(4) $$s_{j}^{max}≜\frac{d_{j}}{p_{j}}$$

In this manner, the deadline $d_{j}$ is, in any case, larger than the expanded period, i.e., $d_{j}\geq s_{j}p_{j}$.

In Table 1 we report a summary of all the symbols used in this work. The considered problem is then to allocate the *k* requests to the *n* available things so as to minimize the maximum energy consumption rate per thing, whilst guaranteeing that all service invocations of applications’ requests are served within their deadline $d_{j}$. 

Formally:

(5) $$\underset{}{\min}\left(\underset{i\in N}{\max}\underset{j\in K}{\sum }\frac{1}{\sum_{i\in N}x_{ij}}f_{ij}x_{ij}\right)$$

s.t.

(6) $$\underset{i\in N}{\sum }x_{ij}\geq 1,\;j\in K$$

(7) $$\underset{i\in N}{\sum }x_{ij}\leq \frac{d_{j}}{p_{j}},\;j\in K$$

(8) $$\underset{j\in K}{\sum }\frac{u_{ij}}{\sum_{i\in N}x_{ij}}x_{ij}\leq v_{i},\;i\in N$$

(9) $$x_{ij}\in \left\{0,1\right\},\;i\in N,j\in K$$

where:

(10) $$x_{ij}=\{\begin{array}{c} 1\;\;\;\\ 0\;\;\; \end{array}\begin{array}{c} if\;request\;j\;is\;allocated\;to\;thing\;i\\ otherwise \end{array}$$

Constraint (6) ensures that each service request is mapped to at least one thing. Constraint (7) limits the split factor of each request to its maximum $s_{j}^{max}$. Note that, based on the above definition, the split factor $s_{j}$ is given by:

(11) $$s_{j}=\underset{i\in N}{\sum }x_{ij}$$

Constraint (8) ensures the schedulability of all the requests (partially) mapped to each thing. Since the deadline $d_{j}$ of (the fraction of) service invocations of request j mapped to thing i is larger than the (extended) period $s_{j}p_{j}$ with which they arrive at the thing, a sufficient condition for the schedulability of the requests is provided by the well-known rate monotonic bound on the thing utilization, defined as:

(12) $$v_{i}=a_{i}\left(2^{\frac{1}{a_{i}}}-1\right)$$

where $a_{i}$ is the number of requests allocated to the thing i, i.e.,:

(13) $$a_{i}≜\underset{j\in K}{\sum }x_{ij}$$

Note that the utilization of the request j to thing i in (8) is calculated by taking the split factor into account. In addition, the definition of $u_{ij}$ in case of $m_{ij}=0$ ensures that no service can be allocated to a thing that does not expose it. 

The problem defined by (5)–(10) is a Mixed Integer Non-Linear problem because of constraint (8), which is non-linear on $x_{ij}$. A relaxed version of this problem can be obtained by assuming that: (i) the deadline of each request is equal to the request’s period, i.e., requests cannot be split across things and thus constraint (6) is equal to 1, and (ii) the schedulability limit is fixed to a constant *v*. The latter, in particular, is a worst-case limit and it is defined by observing that $v_{i}$ is monotonically decreasing with $a_{i}$, which cannot be greater than k. Therefore, for any i, it is $v_{i}\leq v,\;v=k\left(2^{\frac{1}{k}}-1\right)$. The relaxed formulation is a Mixed Integer Linear Problem and, specifically, an instance of an *Agent Bottleneck Generalized Assignment Problem* (ABGAP) that is, in turn, derived by the well-known *Generalized Assignment Problem* (GAP) defined in the literature and known to be NP-hard. Stemming from heuristics proposed to solve GAP and BGAP in [25,26], we designed, in a previous work, a dedicated heuristic [23]. The more general problem defined by (5)–(10), which allows to split requests to multiple things, cannot however be reduced to a linear problem due to the fact that $s_{j}$ is at the denominator of the factors in constraint (8). Moreover, differently from standard ABGAP problems, this problem is based on the assumption that a single task (request) can be assigned to multiple agents (things) and the maximum number of agents for each request is fixed to $s_{j}^{max}$. To the best of our knowledge, there is no well-known general algorithm specifically designed to solve this kind of problem. Starting from the heuristic we proposed in [16], we therefore developed a novel greedy polynomial-time heuristic algorithm that solve the considered problem.

## 5. The MTA Algorithm

In this section we describe the proposed heuristic, named *Multiple Thing Allocation* algorithm (MTA), to solve the problem defined in the previous section. The pseudo-code of MTA is reported in Algorithm 1. The input to MTA are: the number of things n, the number of requests k, the energy consumption rate matrix $F=\left\{f_{ij}\right\}$, the utilization matrix $U=\left\{u_{ij}\right\}$, a precision threshold ε, the max split vector $S^{max}=\left\{s_{j}^{max}\right\}$ and, finally, two different parameters: a desirability matrix D∈NxM and a split policy $S_{p}$. The latter parameters are used to steer the thing allocation procedure *SplitSearch* described in detail below. The output is: a boolean *isFeasable*, which takes the True value if at least one allocation exists, and the allocation matrix Y that maps service requests to things, i.e., each column of Y ($y_{j}$) is the allocation vector for the service request j.


**Algorithm 1:**
*MTA*
**Input**: *n*, *k*, *F*, *U*, *ε*, *S^max^*, *D*, *S_p_* **Output**: *y*, *isFeasible*1:[*y*, *isFeasible*] ← *SplitSearch()*2:if *is Feasible* = *True* then:3:  *last* ← 0; *upper* ← 1; *lower* ← 0;4:  while *upper* – *lower* > *ε* do:5:   *θ* ← (*upper* – *lower*)/26:   [*y*, *isFeasible*] ← *SplitSearch()*7:   if *isFeasible* = *True* then:8:    *last* ← *θ*; *lower* ← *θ*; *θ* ← *θ* + (*upper* – *lower*)/2;9:   else:10:    *upper* ← *θ*; *θ* ← *θ* – (*upper* – *lower*)/2;11:  if *isFeasible* = *False* then:12:   *θ* ← *last*;13:   [*y*, *isFeasible*] ← *SplitSearch()*Pseudo-code of MTA

The rationale behind MTA is to iteratively search for the first feasible allocation that guarantees the highest minimum level of residual battery for all things. To this aim, MTA leverages a procedure SplitSearch that, given a threshold θ, finds an allocation so that the residual battery on each thing after service invocation is no lower than θ, i.e., θ is defined as the minimum residual battery. On every iteration, the threshold θ is decreased until a feasible solution is found with an acceptable precision level measured by ε. More specifically, a binary search strategy is used to reduce the time needed to execute the overall procedure. At any iteration, upper and lower give the current upper and lower bounds on threshold θ, respectively. When the difference between upper and lower is less than the input precision threshold ε, the algorithm stops.

The core of the MTA algorithm is the procedure *SplitSearch* that is reported in Procedure 1. The allocation is based on two input parameters: the desirability matrix D and the split policy $S_{p}$. In particular, each element $d_{ij}$ of D is a measure of the desirability of allocating request j to thing i. The $S_{p}$ parameter is exploited instead by the *SplitPolicy* procedure explained in detail below.

The rationale behind the *SplitSearch* procedure is to iteratively consider all requests and, at each step, select the request that maximize the difference between the largest and the second largest value of the *desirability* matrix (among all the things that can satisfy the request). This means that, on each iteration, the *SplitSearch* algorithm allocates the request that is penalized most if not allocated to the preferred thing. Specifically, in line 7 of Procedure 1 the set $F_{j}$ includes all the things that can satisfy the request (if a thing does not expose a valid service for the request j its $u_{ij}$ is equal to +∞). A thing can satisfy a request if it has enough battery and computational time to serve the request with at least the maximum allowed split factor $s_{j}^{max}$. The actual split $s_{ij}$ for a certain request j on a thing i is computed as the maximum between two ratios: the residual computational capacity and the residual battery (scaled by the minimum residual battery requested θ), both evaluated by assuming that the request j is allocated to the thing i. The value of $s_{ij}$ is a lower bound and means that the thing i is capable of serving the request j if, and only if, the request is split among $s_{ij}-1$ other things. This ensures that all the things in $F_{j}$ do not violate constraint (8) nor the requested minimum residual battery θ. Obviously, if $F_{j}$ is empty the allocation with the requested θ is unfeasible – line 9. 

$F_{j}$ is then exploited to create $S_{j}$ which is a set of sets (line 11). Specifically, $S_{j}$ is composed by t sets with t equal to the cardinality of $F_{j}$ ($t=\left|F_{j}\right|$). Each set, named $S_{ij}$, is composed by the elements of $F_{j}$ that are capable of serving the request j with a split factor lower or equal to $s_{ij}$. Iteratively, for each $i\in F_{j}$ we compute the corresponding minimum split factor $s_{ij}$ and we construct an inner set $\left(S_{ij}\right)$ composed by all the other things that allow the allocation of the request j with, at least, a split $s_{ij}$. As an example, consider the case in which, for a certain j, we obtain $F_{j}=\left\{1,3,6,7\right\}$ with the corresponding $s_{ij}$ vector equal to {2,1,5,1}. The resulting set of sets is: $S_{j}=\left\{S_{0j}=\left\{1,3,7\right\},\;S_{1j}=\left\{3,7\right\},\;S_{2j}=\left\{1,3,6,7\right\},\;S_{3j}=\left\{3,7\right\}\right\}$.


**Procedure 1:**
*SplitSearch*
**Input:**$n,\;k,\;F,\;U,\;\theta ,\;S^{max},\;D,\;S_{p}$ **Output:***Y*, *isFeasible*1:for i←1 **to***n***do**$c_{i}\leftarrow 0,\;e_{i}\leftarrow 0,\;\nu_{i}\leftarrow 1,\;a_{i}\leftarrow 1$2:N←{1, …,n}, K←{1, …, k}3:isFeasible←True4:**while**K≠∅**do:**5:$\delta^{*}\leftarrow -\infty$6:**for each**j∈K**do**:7:$F_{j}=\left\{i\in N:s_{ij}=|max\left(\frac{u_{ij}}{\nu_{i}-c_{i}},\frac{f_{ij}}{\theta -e_{i}})\right|,1\leq s_{ij}\leq s_{j}^{max}\right\}$8:**if**$F_{j}=\emptyset$**then:**9:isFeasible←False10:**return**11:$S_{j}\leftarrow \left\{S_{0j}=\left\{z\in F_{j}:s_{zj}\leq s_{0j}\right\},\;S_{1j}=\left\{z\in F_{j}:s_{zj}\leq s_{1j}\right\},\ldots \;S_{ij}=\left\{z\in F_{j}:s_{zj}\leq s_{ij}\right\}:\forall i\in F_{j}\right\}$12:$S_{j}^{max}\leftarrow \arg\underset{i\in F_{j}}{\max}\left\{\left|S_{ij}\right|:\left|S_{ij}\right|\geq s_{zj},\;\;\;\forall z\in S_{ij}\right\}$13:$i^{max}\leftarrow$$\arg\underset{i\in S_{j}^{\max}}{\max}\left\{d_{ij}\right\}$14:$S_{j}^{max2}\leftarrow \arg\underset{i\in F_{j}\backslash \left\{i^{\max}\right\}}{\max}\left\{\left|S_{ij}\right|:\left|S_{ij}\right|\geq s_{zj},\;\;\;\forall z\in S_{ij}\right\}$15:**if**$S_{j}^{max}=\emptyset$**then:**δ←+∞16:**else:**$\delta \leftarrow d_{i^{max}j}-\underset{i\in S_{j}^{\max_{2}}}{\max}\left\{d_{ij}\right\}$17:**if**$\delta >\delta^{*}$then:$\delta^{*}\leftarrow \delta$; $j^{*}\leftarrow j;$18:**end**19:$\left[z,s_{j^{*}}\right]\leftarrow SplitPolicy\left(S_{p},\;S_{j^{*}}^{max},\;\;\;i^{max},\;\;S_{j^{*}}\right)$20:**for each**$i^{*}\in z$**do:**21:$c_{i^{*}}\leftarrow c_{i^{*}}+u_{i^{*}j^{*}}/s_{j^{*}}$; $e_{i^{*}}\leftarrow e_{i^{*}}+f_{i^{*}j^{*}}/s_{j^{*}}$; $a_{i^{*}}\leftarrow a_{i^{*}}+1$; $\nu_{i^{*}}\leftarrow a_{i^{*}}\left(2^{\frac{1}{a_{i^{*}}}}-1\right)$; $Col_{j^{*}}\left(Y\right)\leftarrow z$;22:**end**23:$K\leftarrow K/\left\{j^{*}\right\}$24:**return**YPseudo-code of *SplitSearch.*

Based on $S_{j}$ the algorithm computes $S_{j}^{max}$ (line 12) which is defined as the index of $S_{ij}$ with the maximum cardinality among $S_{ij}$ *valid* sets. A set is *valid* if the maximum $s_{ij}$, within the set, is lower or equal to the its cardinality $\left|S_{ij}\right|\leq s_{zj},\;\forall z\in S_{ij}$. Considering the previous example, the resulting $S_{j}^{max}$ is 0 (the index of $S_{0j}$) because the set $S_{2j}$ is not valid due to the fact that the thing with index 6 requires an $s_{ij}=5$ (the request must be split among, at least, 5 things) but the cardinality of its set is $\left|S_{2j}\right|=4$ (only 4 things are available to serve the request). 

$S_{j}^{max}$ is then exploited to derive $i^{max}$ that is the index of the thing, within $S_{j}^{max}$, with the largest value in the *desirability* matrix – see line 13. As already pointed out, however, the rationale behind the *SplitSearch* algorithm is to allocate the request that maximizes the difference between the largest and the second largest $d_{ij}$. For this reason, in line 14 the algorithm computes $S_{j}^{max2}$ obtained exactly in the same way as $S_{j}^{max}$, except for the fact that the maximum is evaluated by removing $i^{max}$ from $F_{j}$. The difference between the two values of $d_{ij}$ is evaluated in line 16. Specifically, the algorithm has an internal variable δ used to find, at each iteration, the $j^{*}$ that maximize the difference between the largest and second largest $d_{ij}$ among all the requests not yet allocated. 


**Algorithm 2:**
*SplitPolicy*

1:
**if** $S_{p}=MaxSplit$ **then:**
2:$i\leftarrow S_{j^{*}}^{max}$; $s_{j^{*}}\leftarrow \left|S_{ij^{*}}\right|$; $z\leftarrow S_{ij^{*}};$3:
**else if** $S_{p}=MinSplit$ **then:**
4:$i\leftarrow i^{max}$; $s_{j^{*}}\leftarrow \left|S_{ij^{*}}\right|$; $z\leftarrow S_{ij^{*}}$;5:
**else if** $S_{p}=NoSplit$ **then:**
6:$s_{j^{*}}\leftarrow 1$; $z\leftarrow i^{max}$;7:
**return** $s_{j^{*}},\;z$

Pseudo-code of *SplitPolicy.*

At the end of the inner loop, in line 19, the algorithm calls the *SplitPolicy* procedure that, based on the chosen $j^{*}$ request, selects the actual split $s_{j^{*}}$ by adopting one of the three available policies: (i) *Maximum Split*, (ii) *Minimum Split* and (iii) *No Split*. Specifically, the *Maximum Split* policy always tries to split the request among the maximum number of available things, whereas the *Minimum Split* policy always selects the smaller split factor $s_{j^{*}}$ that allows the request to be served. Finally, the *No Split* policy does not allow any split for any request ($s_{j^{*}}=1,\;\forall j\in K$). 

**Figure 3:**
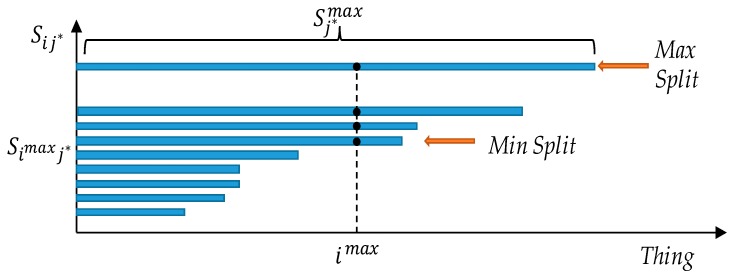
*SplitPolicy* example.

The *SplitPolicy* procedure is reported in Algorithm 2. The Maximum Spilt is achieved by dividing the request $j^{*}$ between the $S_{ij^{*}}$ things with $i=S_{j^{*}}^{max}$ (line 2). On the other hand, the Minimum Split is achieved by selecting the minimum split that includes the thing $i^{max}$, identified by $S_{i^{max}j^{*}}$ (line 4). Finally, for the No Split policy only, the $i^{max}$ thing is selected (line 6). It is worth to note that, regardless of the adopted policy, the $i^{max}$ thing is always selected. To better describe this behavior we rely on Figure 3 where we report the set $S_{j^{*}}$ ordered by the cardinality of its inner sets $S_{ij^{*}}$. Specifically, on the x axis we report the index of all the things, whereas on the y axis we report the $S_{ij^{*}}$ sets ordered by their cardinality. The Minimum Split is identified by the first set that includes $i^{max}$. Because of the ordering, all the sets above will also include $i^{max}$, i.e., if a thing i can serve a request with $s_{j}=x$, it can also serve the same request with any other split factor larger than x. The Maximum Split is, therefore, the one with cardinality equal to $S_{j^{*}}^{max}$. If two sets $S_{ij^{*}}$ have the same cardinality, then the desirability of the things within the two sets is considered.

Once the allocation is performed the *SplitSearch* procedure continues and updates the costs of all the selected things, lines 21 of Procedure 1. Finally, the request $j^{*}$ is removed from the set K and the procedure restarts. At the end, the resulting allocation matrix Y is returned.

Several approaches to set the *desirability matrix* are possible. However, based on some preliminary tests, we determined that good results are obtained by considering the three cases D=F, D=−F and D=U, that correspond to giving a higher preference to requests with the *largest energy cost*, the *lowest energy cost*, and the *largest computational cost*, respectively. The best configuration, however, depends on the specific scenario with its energy and computational costs. For this reason, we run the algorithm three times, one for each *desirability matrix* configuration, and we choose the configuration that yields the best result.

The computational complexity of the MTA algorithm is $O\left(\alpha \beta \left(nk^{3}\right)\right)$.

**Proof.** Let us first evaluate the complexity of the SplitSearch procedure. The most expensive phase, on each iteration, is the computation of $F_{j}$ and $S_{j}$ which both requires O(nk) time, i.e., in the worst case all things (n) can serve all requests (k). To compute $S_{j}^{max}$ and $S_{j}^{max2}$ the algorithm needs O(nk). The same is valid for finding the first and the second $i^{max}$. The while loop (line 4) performs O(k) assignments, hence by considering also the inner loop (line 6) we obtain a total of $O\left(k^{2}\right)$ time. We can conclude that the overall time complexity is $O\left(nk^{3}\right)$. The complexity of MTA is governed by the choice performed on ε. We can derive β according to:

(14) $$\beta =\log_{2}\frac{upper-lower}{\varepsilon }$$

whereas, in our case, α is fixed to three (α=3) because of the three considered desirability matrix: highest energy cost, lowest energy cost and highest computational cost, respectively. Hence, the overall complexity is $O\left(\alpha \beta \left(nk^{3}\right)\right)$. ■

Several optimizations have been carried out to reduce the time complexity in the average case. Among the others, in the actual implementation we sort the things in $F_{j}$ according to the maximum allowed split. To build $S_{j}$ we iteratively scan $F_{j}$ producing an inner set $S_{ij}$ on each iteration. Thanks to the ordering of $F_{j}$, each inner set $S_{ij}$ is, therefore, composed by the things of $F_{j}$ ordered according to the maximum allowed split. Moreover, by following the iterative approach, the resulting $S_{j}$ is also ordered according to the cardinality of each inner set, thus the complexity of finding the largest and second largest element of $S_{j}$ is reduced. In Figure 3 we already reported a graphical representation of an ordered $S_{j}$, that reflects the optimization we designed. Indeed, we can find $S_{j}^{max}$ by iteratively searching from the last thing within the last inner set of $S_{j}$. Recalling that a split associated to a thing, say $s_{ij}$, is valid only if there are at least other $s_{ij}$ things that allow a split lower or equal to $s_{ij}$, we can start from the last thing in the last inner set of $S_{j}$ and stop whenever we find a thing i in position x (within its inner set) with $x\geq s_{ij}$. That is, if the position, say x, of a thing within the ordered inner set $S_{ij}$ is larger than the associated split factor, then there are at least x other things that can accommodate the request j with a split factor less or equal to $s_{ij}$ (in Figure 3, by design, all the things on the left side of a thing i must have a split factor <= $s_{ij}$). With respect to the previous example with $F_{j}=\left\{1,3,6,7\right\}$ and the corresponding $s_{ij}$ vector equal to {2,1,5,1}, the resulting ordered $F_{j}$ is redefined as $F_{j}=\left\{3,7,1,6\right\}$. Therefore $S_{j}=\left\{S_{0j}=\left\{3,7\right\},\;S_{1j}=\left\{3,7\right\},\;S_{2j}=\left\{3,7,1\right\},\;S_{3j}=\left\{3,7,1,6\right\}\right\}$. We can find the largest and second largest element of $S_{j}$ ($S_{j}^{max}$ and $S_{j}^{max2}$) by iteratively searching from the end of the order set $S_{j}$. The first thing analyzed is 6 that is in position x=4 within $S_{3j}$ but it requires $s_{ij}=5$ ($x<s_{ij}$), thus the next inner set is analyzed. The next thing is 1 which requires $s_{ij}=2$ and it is in position x=3 ($x\geq s_{ij}$) thus $S_{j}^{max}=S_{2j}$.

## 6. Performance Evaluation

In this section we evaluate the proposed algorithm MTA by means of a set of experiments in different scenarios. Specifically, we implemented our algorithm in Java and we run the experiments on a machine with a Linux 64bit operating system. The machine is equipped with an Intel® Core™ i7-4770 CPU @ 3.40 GHz, and 16 GB of RAM. The parameters used in our experiments are reported in Table 2, where the values are uniformly distributed across the presented ranges. We vary the number of things n, the number of requests k and also the average number of things available to serve each request, called *ratio*, expressed as a fraction of the overall number of things. To this aim, $m_{ij}$ and $d_{j}$ are randomly generated in each experiment uniformly so as to meet the target *ratio* of the experiment.

Each experiment has been generated to test all the available split policies. To this aim, deadlines have been set to always allow the maximum split, which is thus controlled by the experiment’s ratio. Moreover, each problem instance has been generated to have at least one feasible solution. For each experiment, one hundred different problem instances are generated. The value of the objective function calculated by (5), i.e., the maximum energy consumption rate per thing achieved by the solution computed by the MTA algorithm, is then reported along with 95% confidence interval.

We evaluate the MTA algorithm by defining four different scenarios, characterized by four different *ratios*: 5%, 25%, 50% and 75%, respectively. Indeed, the proposed MTA algorithm can be exploited in almost all scenarios, from small-scale IoT systems, characterized by smaller ratios, to massive IoT deployments with a huge number of equivalent things. However, it is worth to note that we consider also IoT deployments with a very large ratio equal to 75%. Even if such scenarios are less common, we aim at challenging the proposed solution also in scenarios that are more difficult to solve. In fact, the larger the ratio is, the largest the solution space. Within each scenario, we vary the number of requests from 40 to 100, whereas the number of things is fixed across each experiment. Numerical experiments have been conducted with several combinations of requests and things. Results are similar in all considered experiments, therefore, without lack of generality, we only report the results obtained with 50 things. 

**Figure 4:**
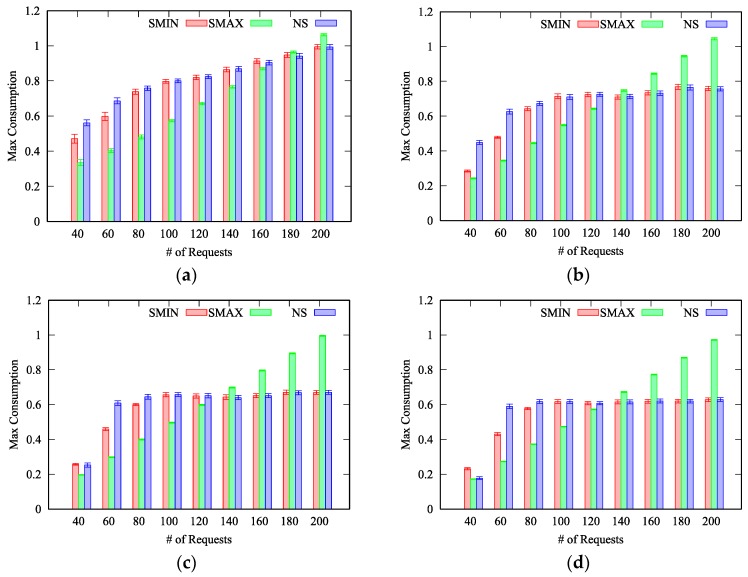
Max consumption: (**a**) Ratio 5%; (**b**) Ratio 25%; (**c**) Ratio 50%; (**d**) Ratio 75%.

In Figure 4 we report the average maximum consumption obtained with 50 things when the ratio is 5% (Figure 4a), 25% (Figure 4b), 50% (Figure 4c) and 75% (Figure 4d), for the three different split policies Maximum Split (*SMAX*), Minimum Split (*SMIN*) and No Split (*NS*), respectively. Obviously, if the number of things is fixed, the maximum consumption rate increases with the number of requests to be allocated. As can be seen, the minimum maximum consumption is obtained with a different split policy depending on the number of things, the number of requests and the ratio. 

**Figure 5:**
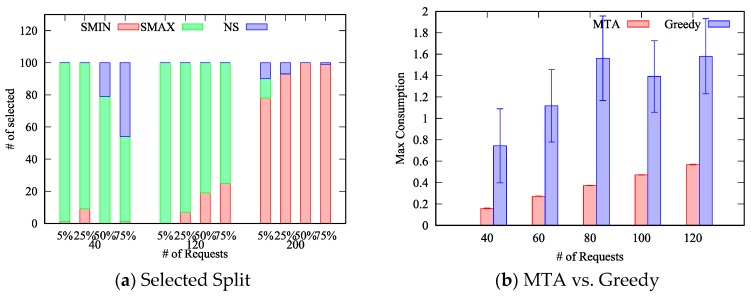
Split Policy and MTA vs. Greedy.

To better clarify this aspect, in Figure 5a we report the number of times a specific split policy has been selected as the best one. We can notice that when there are few requests (k≤120) compared to the number of things, the *SMAX* policy achieves the best performance in most of the cases. Indeed, by splitting across multiple things, intuitively each thing is less loaded and thus the maximum energy consumption is reduced. On the other hand, when the number of requests further increases (k≥140), the *SMIN* policy achieves the best result because of the rate monotonic constraint. Specifically, each request has computational and energy costs that are split among all the things to which the request is allocated. However, both the computational and energy costs are not the same across all things, e.g., some things may consume more energy than others to handle the same request. When there are many requests to be allocated, and all the requests are split between the maximum number of things, the probability that certain requests cannot be allocated to their preferred things (derived from the *desirability matrix*) increases significantly. Specifically, such things may be already in charge of handling other requests (or at least parts of them) and their utilization may be already at the maximum allowed value. In such cases, the minimum split policy performs better because, at each iteration, it selects a smaller set of things, i.e., the smallest set that includes the preferred thing for the specific request, thus leaving more space to map next requests to other things. It therefore follows that the probability for a request to find the preferred thing with its utilization already at the maximum value is lower because it is less likely, with respect to the *SMAX* policy, that other requests have been allocated to the preferred thing as part of the *SplitPolicy* procedure. 

It is worth to note that, in less dense deployments, characterized by a ratio around 5%, the *SMAX* policy achieves the best result also when the number of requests increases (k=160) – see Figure 4a. Such behavior can be explained considering that, on each allocation, the maximum set of preferred things is limited by the small ratio, and therefore the probability of finding the preferred thing with its utilization already at the maximum value is reduced in comparison to scenarios characterized by larger ratios.

Furthermore, we can notice that the *NS* policy achieve results that are very close to the solution found by the *SMIN* policy. This can be explained considering that the *NS* policy is a particular case of the *SMIN* policy. Specifically, when the set including the preferred thing is composed only by the preferred thing, the two policies behave exactly in the same manner. Based on our results, reported in Figure 4, we can conclude that the *NS* policy performs worst or equal to the *SMIN* policy in most of the cases, with only two exceptions: with 40 requests and a ratio equal to 50% and 75%. Therefore, when time complexity is a concern, the *NS* policy can be omitted without affecting the obtained best results in almost all cases. It therefore derives that the split of requests between multiple things, compared to the one-to-one mapping, can effectively reduce the energy consumption in almost all the considered scenarios, and in particular in less dense IoT deployments. In fact, in Figure 5a we can notice that, for the scenario with a ratio equal to 5%, the *SMAX* policy has been always selected even with k=120, whereas with ratios equal to 25%, 50% and 75%, respectively, some of the best results are achieved by the *SMIN* policy.

Finally, we compare our *MTA* algorithm against a *greedy* algorithm designed to always allocate requests to the thing that maximizes the *desirability matrix* in the three cases D=F, D=−F and D=U, and then selecting the best result as the solution. This experiment has been designed to estimate the advantages of our solution compared to a “standard” QoS-aware algorithm. In Figure 5b, we report the comparison between *MTA* and the *greedy* algorithm for 50 things with a ratio of 75%. As can be seen, *MTA* always outperforms the *greedy* algorithm in all the considered scenarios. In particular, the resulting maximum energy spent by the most energy-consuming thing with the *MTA* allocation is between a quarter to half of the maximum energy consumed with the *greedy* allocation, i.e. the lifetime of the system is twice to for times longer. Moreover, the *MTA* algorithm shows a very regular behavior around the average since the variability of the result, measured by the confidence interval, is very small, whereas the *greedy* algorithm is affected by large fluctuations between different instances of the problem. This can be explained considering two different aspects: (i) the *MTA* algorithm uses the most efficient split policy, and (ii) the *MTA* algorithm allocates requests based on the difference between the largest and second largest value of the *desirability matrix*. The former aspect allows *MTA* to distribute the energy cost among multiple things, the second one, instead, allows *MTA* to consider the impact of an allocation on the other requests. The *greedy* algorithm, instead, does not exploit such techniques: requests that have highly heterogeneous costs between things may be drastically penalized. 

Based on these results, we can notice that the split of requests among different things in consecutive periods can effectively reduce the maximum energy consumption. Specifically, the *MTA* algorithm always selects the best solution, among all the split policies and all the value of the desirability matrix, in order to adapt to different scenarios, each one characterized by a different number of both requests and things. 

## 7. Conclusions

In this work we proposed a solution to allocate QoS-aware requests in a massive IoT deployment. We formally defined the problem along with a detailed QoS characterization of the IoT systems and all its building blocks. Then, we proposed an efficient heuristic, which fully exploits the context information in order to maximize the lifetime of the connected IoT systems. Finally, we evaluated our proposed solution through simulation and compared the obtained results against a greedy algorithm, showing how the proposed solution fully exploits the context information to effectively reduce the power consumption among things. Based on our results, we foresee that the MTA algorithm can be exploited to enhance available IoT-Cloud solutions by effectively splitting requests to multiple available smart devices. As a matter of facts, the FIWARE architecture relies on a particular GE, called Orion Context Broker, that is a publish/subscribe context broker. Smart devices publish their values enriched with context information, whereas applications can retrieve stored values from the broker by means of context-enriched queries. The MTA algorithm can be easily integrated within the Orion Context Broker, or by means of a dedicated IoT Broker (https://forge.fiware.org/plugins/mediawiki/wiki/fiware/index.php/FIWARE.OpenSpecification.IoT.Backend.IoTBroker), to resolve application queries to different things in order to extend the lifetime of connected things. 

Moreover, especially when the fog layer is employed, our solution, in conjunction with a monitoring framework, can be exploited to handle the dynamic nature of IoT deployments by reallocating requests at runtime whenever a change in the environment occur. Such behavior is of paramount importance in scenarios characterized by real-time requirements in which the edge layer is exploited to aggregate and process data in timely manner. The FIWARE IDEC GE (https://fimac.m-iti.org/5d.php), for example, has been designed to aggregate data on a gateway and to implement a Complex Event Processing engine that allows applications to only subscribe to value-added data. The MTA algorithm can be exploited to drive the gathering of important data allowing to meet application requirements whilst reducing the overall energy consumption.

## Figures and Tables

**Table 1:** Used symbols.

Symbol	Description
N	Set of connected things with cardinality n
i	Thing *i*
K	Set of incoming requests with cardinality k
j	Request *j*
$b_{i}$	Available energy of thing *i*
$p_{j}$	Period of request *j*
$d_{j}$	Deadline of request *j*
M	Context matrix
$m_{ij}$	Elements of the context matrix
$t_{ij}$	Execution time of request *j* on thing *i*
$c_{ij}$	Energy cost of request *j* on thing *i*
U	Utilization matrix
$u_{ij}$	Utilization of request *j* on thing *i*
F	Normalized cost matrix
$f_{ij}$	Normalized cost of request *j* on thing *i*
$s_{j}$	Split factor for request *j*
$s_{j}^{max}$	Maximum split factor for request *j*
$v_{i}$	Rate monotonic schedulability limit
$a_{i}$	Number of requests allocated to thing i

**Table 2:** Simulation parameters.

Parameter	Value Range
$f_{ij}$	0.001−0.5
$u_{ij}$	$10^{-4}-10^{-3}$
n	30–150
k	30–100
*ratio*	5%, 25%, 50%, 75%
*Split policy*	*SMAX*, *SMIN*, *NS*
